# Physiological and Metabolic Responses Triggered by Omeprazole Improve Tomato Plant Tolerance to NaCl Stress

**DOI:** 10.3389/fpls.2018.00249

**Published:** 2018-02-27

**Authors:** Youssef Rouphael, Giampaolo Raimondi, Luigi Lucini, Petronia Carillo, Marios C. Kyriacou, Giuseppe Colla, Valerio Cirillo, Antonio Pannico, Christophe El-Nakhel, Stefania De Pascale

**Affiliations:** ^1^Department of Agricultural Sciences, University of Naples Federico II, Portici, Italy; ^2^Department for Sustainable Food Process, Università Cattolica del Sacro Cuore, Piacenza, Italy; ^3^Department of Environmental, Biological and Pharmaceutical Sciences and Technologies, University of Campania “Luigi Vanvitelli”, Caserta, Italy; ^4^Department of Vegetable Crops, Agricultural Research Institute, Nicosia, Cyprus; ^5^Department of Agricultural and Forestry Sciences, University of Tuscia, Viterbo, Italy

**Keywords:** benzimidazole, gas exchange, hormone-like activity, ion homeostasis, metabolomics, salt stress, *Solanum lycopersicum* L

## Abstract

Interest in the role of small bioactive molecules (< 500 Da) in plants is on the rise, compelled by plant scientists' attempt to unravel their mode of action implicated in stimulating growth and enhancing tolerance to environmental stressors. The current study aimed at elucidating the morphological, physiological and metabolomic changes occurring in greenhouse tomato (cv. Seny) treated with omeprazole (OMP), a benzimidazole inhibitor of animal proton pumps. The OMP was applied at three rates (0, 10, or 100 μM) as substrate drench for tomato plants grown under nonsaline (control) or saline conditions sustained by nutrient solutions of 1 or 75 mM NaCl, respectively. Increasing NaCl concentration from 1 to 75 mM decreased the tomato shoot dry weight by 49% in the 0 μM OMP treatment, whereas the reduction was not significant at 10 or 100 μM of OMP. Treatment of salinized (75 mM NaCl) tomato plants with 10 and especially 100 μM OMP decreased Na^+^ and Cl^−^ while it increased Ca^2+^ concentration in the leaves. However, OMP was not strictly involved in ion homeostasis since the K^+^ to Na^+^ ratio did not increase under combined salinity and OMP treatment. OMP increased root dry weight, root morphological characteristics (total length and surface), transpiration, and net photosynthetic rate independently of salinity. Metabolic profiling of leaves through UHPLC liquid chromatography coupled to quadrupole-time-of-flight mass spectrometry facilitated identification of the reprogramming of a wide range of metabolites in response to OMP treatment. Hormonal changes involved an increase in ABA, decrease in auxins and cytokinin, and a tendency for GA down accumulation. Cutin biosynthesis, alteration of membrane lipids and heightened radical scavenging ability related to the accumulation of phenolics and carotenoids were observed. Several other stress-related compounds, such as polyamine conjugates, alkaloids and sesquiterpene lactones, were altered in response to OMP. Although a specific and well-defined mechanism could not be posited, the metabolic processes involved in OMP action suggest that this small bioactive molecule might have a hormone-like activity that ultimately elicits an improved tolerance to NaCl salinity stress.

## Introduction

Salinity affects more than 45 million hectares (20%) of irrigated soils accounting for one-third of worldwide food production (Machado and Serralheiro, 2017). In Europe, about 4 million hectares have been impoverished by human activities, in particular along the Mediterranean coast (Daliakopoulos et al., 2016). Climate change, rise in evapotranspiration, intensive farming, excessive over-pumping of groundwater for irrigation (especially in coastal areas with consequent sea-water infiltration into fresh aquifers) and use of low quality water (brackish water or treated wastewater) in irrigation contribute synergically to soil salinization (Rana and Katerji, 2000; Costantini and Lorenzetti, 2013; Daliakopoulos et al., 2016). Under these circumstances, continuous exposure to hyperosmotic stress and seasonal effects linked to salt accumulation in the roots highly affect crop yield (Rana and Katerji, 2000).

Osmotic stress and ion toxicity are the main problems that affect salt stressed plants (Munns and Tester, 2008; Gorham et al., 2010). Under high salinity, roots are unable to uptake water from the soil and toxic concentrations of sodium and chloride build up in the cytosol and organelles, resulting in plant nutritional disorders and oxidative stress (Hasegawa et al., 2000; Munns, 2002; Tavakkoli et al., 2010). Sodium interferes with potassium and calcium uptake, negatively affecting stomatal control; moreover, it can replace potassium in key enzymatic reactions. Therefore, the salt stress status of a crop depends mainly on the potassium-to-sodium ratio than on the absolute amount of sodium in the cytosol (Shabala and Cuin, 2008; Asins et al., 2013; Annunziata et al., 2017). Instead, chloride competes with nitrate for uptake and translocation within the plants by nitrate transporter proteins, exerting direct and indirect effects mediated by nitrate decrease on chlorophyll degradation as well as on the PSII quantum yield and photochemical quenching (Carillo et al., 2005; Tavakkoli et al., 2011). This double effect reduces plant growth and causes irreversible cell damage. However, plants try to adapt to salinity by osmo-regulating cellular compartments and controlling ion and water homeostasis to reduce stress damage and resume growth (Hasegawa et al., 2000; Woodrow et al., 2017). In particular, a ubiquitous mechanism of plant cells involves compartmentalization of toxic ions in the vacuoles as inexpensive osmotica and synthesis and/or accumulation of organic osmolytes in the cytosol for osmotic adjustment and protection against oxidative stress (Carillo et al., 2008; Hasegawa, 2013; Shabala, 2013). In this important process, plasma membrane and vacuolar H^+^-ATPases have a key role in cytosol detoxification by creating an electrochemical H^+^ gradient across the membranes used to drive a secondary active transport for Na^+^ compartmentalization within the vacuole or its extrusion from the cell (Blumwald et al., 2000; Pardo et al., 2006; Ji et al., 2013). In fact, it is generally accepted that salt stress induces H^+^-pumping capacity in plant tissues, mainly to energize Na^+^/H^+^ exchanger activity (Cuin et al., 2011; Bose et al., 2015). Moreover, the electrochemical gradient built up can be channeled for driving the active co-transport with H^+^ of nitrate, phosphate, sulfate, sucrose, hexoses, and amino acids against their gradient (Batelli et al., 2007; Silva and Gerós, 2009; Conde et al., 2011).

Proton pump activity is continuously modulated by all the important factors controlling the plant physiology, subject to activation/deactivation foremost in response to abiotic stresses (Chelysheva et al., 1999; Hasegawa, 2013). Salt tolerance in *Arabidopsis* is enhanced as a result of increased ion compartmentalization facilitated by overexpressing vacuolar H^+^-PPase AVP1 (Fuglsang et al., 2011) and, furthermore, by co-overexpressing vacuolar H^+^-PPase*AVP1* and Na^+^/H^+^ antiporter *AtNHX1* genes simultaneously (Shen et al., 2015). Conversely, inhibition of plasma membrane H^+^-ATPase by vanadate decreases the K^+^/Na^+^ ratio rendering the plant more susceptible to salinity (Li et al., 2014).

Homologues of plant proton pumps are the gastric H^+^/K^+^-ATPases, members of the P2-type ATPase family, responsible for gastric acid secretion, which include also membrane Ca^2+^ pumps and Na^+^/K^+^-transporters (Shin et al., 2009). The introduction and use of substituted benzimidazoles as proton pump inhibitors (PPI) targeted to the gastric H^+^/K^+^-ATPases has been essential for the treatment of peptic ulcers and gastroesophageal reflux disease (Fellenius et al., 1981). Omeprazole (OMP) has been the first PPI pharmaceutical introduced in the market, which specifically and irreversibly inhibits the P2-type ATPases (Shin and Kim, 2013). It is thus used for the treatment of dyspepsia, peptic ulcer, gastroesophageal reflux disease or *Helicobacter pylori* infection (Seoane et al., 2017).

Over the past few decades, plant scientists have started to identify the targets and mode of action in plants of signaling small molecules (< 500 Da) derived from human/animal research (Kaschani and van der Hoorn, 2007; Lace and Prandi, 2016). These small bioactive molecules created on the basis of natural or synthetic low-molecular weight compounds could be considered an efficient and safe approach to stimulate plant growth and elicit tolerance to environmental stressors (Kaschani and van der Hoorn, 2007; Lace and Prandi, 2016; Tsygankova et al., 2016).

Notwithstanding P2-type ATPases are not present in plants, Van Oosten et al. (2017) have demonstrated OMP (345.4 Da) as being effective at micromolar (μM) concentrations in stimulating tomato plant growth and enhancing tolerance to salinity. However, the experiments discussed by Van Oosten et al. (2017) pertained to a short term trial, while in horticultural context plants growing on saline soils usually encounter long-term sodium chloride salinization. Moreover, though the effectiveness of OMP in inducing stress tolerance has been partially clarified, conclusive evidences regarding its molecular targets have not become available yet. Nonetheless, it is important to unravel the molecular basis of the improved stress tolerance imparted by OMP treatments, in order to elucidate the physiological and biochemical mechanisms involved, thus supporting a rationale for their application in agriculture. In this context, an untargeted approach facilitated by metabolomics has proved a powerful strategy for shedding light onto the role of secondary metabolites in mediating plant response to abiotic stressors (Nakabayashi and Saito, 2015).

Indisputably, the elucidation of fundamental plant molecular responses to OMP can be instrumental to unraveling adaptive strategies against salinity stress; hence the aim of this study was to investigate morphological, physiological, and metabolic changes in response to OMP application onto greenhouse tomato subjected to salt stress conditions. Untreated and treated tomato plants were characterized and compared in terms of growth, root morphology, ion content, gas exchange parameters, water relations and metabolic profiling.

## Materials and methods

### Plant material, greenhouse conditions, and crop management

The experimental trial was carried out in the 2016 summer season in an unheated glasshouse at the experimental station of the University of Naples Federico II, located in Bellizzi, Salerno province (43° 31′ N, 14° 58′ E; 60 m asl), Italy. The tested vegetable species for the current experiment was tomato (*Solanum lycopersicum* L.) cv. Seny (Seminis Monsanto, Milano, Italy). Tomato plant were grown under natural light conditions and the daily air temperature inside the glasshouse was maintained between 18 and 30°C.

Cultivar Seny is a round-fruited, indeterminate tomato vine widely cultivated under greenhouse conditions in Italy due to its high productivity and resistance to cracking. Tomato seedlings were transplanted on May 2, at the three-true-leaf phenological stage into plastic pots (h 20 cm; d 20 cm) containing 5.3 L of a peat/perlite mixture in 2:1 volume ratio. The Lithuanian peat containing sphagnum peat moss (Agraria Di Vita, Pistoia, Italy) had the following physicochemical properties: 80% water holing capacity, pH 4.0, electrical conductivity 0.1 dS m^−1^, 11 g kg^−1^ N, 0.1 g kg^−1^ P, 0.1 g kg^−1^ K, 1.8 g kg^−1^ Ca, 2.0 g kg^−1^ Mg, 70 mg kg^−1^ Fe, 15 mg kg^−1^ Mn, and 4 mg kg^−1^ Zn. Plastic pots were arranged in double rows. Plant rows were 0.9 m apart, and the space between plants within a row was 0.3 m. The distance between the centers of double rows was 2.22 m, resulting in a plant density of 3 plants m^−2^, as normally practiced among fresh tomato greenhouse growers. Throughout the cultural cycle pathogens and pests were controlled based on standard phytoprotective practices used by commercial tomato growers in Italy.

### Experimental design, omeprazole application, and nutrient solution management

The experiment was designed as a two-way factorial design encompassing combinations of two sodium chloride (NaCl) concentrations (1 mM nonsaline control and 75 mM NaCl) in the nutrient solution and three omeprazole (OMP) application levels (0 control, 10 and 100 μM OMP). The treatments were arranged in a randomized complete-block design with four replicates, amounting to a total of 24 experimental units with four plants each (*n* = 96 plants). The OMP was applied as substrate drench treatment five times during the growing cycle at weekly intervals starting on 10 May (9 days after transplanting; DAT). All OMP applications were delivered at a uniform rate of 100 mL per plant.

The basic (nonsaline) nutrient solution had the following composition: 13.6 mM N-NO_3_, 2.0 mM S, 1.4 mM P, 6.0 mM K, 4.5 mM Ca, 2.0 mM Mg, 1 mM Na, 1 mM Cl, 20 μM Fe, 9 μM Mn, 1.5 μM Cu, 3 μM Zn, 20 μM B, and 0.3 μM Mo with an electrical conductivity (EC) of 2.0 dS m^−1^. The saline nutrient solution treatment consisted of the same basic composition plus an additional 75 mM NaCl, yielding an EC value of 9.2 dS m^−1^. The pH of the two nutrient solutions was 6.2 ± 0.3. The nonsaline and saline nutrient solutions were prepared using deionized water. Saline treatment was initiated on May 18 (17 DAT).

The nutrient solution was pumped from independent tanks and delivered through a drip irrigation system with one emitter per plant at a flow rate of 2 L h^−1^. All plants received the same amount of solution with a leaching fraction of 20% to avoid build up of salinity into the substrate. A leaching fraction of 20% is needed to maintain the EC in the substrate to a similar level to the nutrient solution EC (Colla et al., 2012, 2013).

### Yield, growth measurements, and root characteristics

The number of fully ripe fruits as well as the fresh weight of marketable fruit of the first two trusses were recorded on all plants. At the end of the experiment (July 5, 65 DAT), plants were separated into leaves, stems and roots. All plant tissues were dried at 80°C for 72 h until they reached a constant weight which corresponded to their dry biomasses. Shoot dry weight was equal to the sum of the aerial vegetative parts (leaves + stems), and the root-to-shoot ratio was also calculated. Dried plant tissues were sampled for ion analyses. The total leaf area per plant was measured using an electronic area meter (Li-Cor3000, Li-Cor, Lincoln, NE, USA).

The plant height as well as the number of leaves per plant were counted. Also, the root system architecture components were determined. Root system collection and sample preparation were performed following the protocol described previously by Lucini et al. (2015) and Rouphael et al. (2017a). The determination of root morphology characteristics was performed using WinRHIZO Pro (Regent Instruments Inc., Canada), connected to an image analysis scanner (STD 4800 Regent Instruments Inc., Canada). Three-dimensional images were captured and the following root characteristics were determined: root diameter, total root length and surface.

### Leaf water potential, relative water content, and leaf gas exchange measurements

On June 6 (36 DAT), leaf water potential (Ψ_l_) measurements were performed on three replicates per treatment, using a dew-point psychrometer (WP4; Decagon Devices, Pullman, WA). The Relative Water Content (RWC) of basal and apical tomato leaves was calculated following the formula described by Jones and Turner (1978) (RWC = [FW−DW]/[TW−DW] × 100); where FW, DW and TW corresponded to fresh, dry and turgid weight, respectively.

At 58 DAT, the net CO_2_ assimilation rate (A_CO2_), stomatal resistance (r_s_) and transpiration rate (E) were measured with a portable gas exchange analyzer (LCA-4; ADC BioScientific Ltd., Hoddesdon, UK) equipped with a broadleaf chamber (cuvette window area, 6.25 cm^2^). This measurement was carried out within 2 h across solar noon (i.e., between 11.00 and 13.00) on the youngest fully expanded leaves, using six replicates for each treatment. Photosynthetically active radiation (PAR), Relative humidity (RH) and CO_2_ concentration (593 ± 8 μmol m^−2^ s^−1^, RH 50 ± 0.6% and 377 ± 0.6 mg kg^−1^, respectively) were set at ambient value and the flow rate of air was 400 mL s^−1^. The Water Use Efficiency (WUE) was calculated as A_CO2_/E.

### Ion analyses

Dried plant tissues (leaf, fruit, and root) were ground separately in a Wiley mill (IKA, MF10.1, Staufen, Germany) to pass through 0.5 mm sieve, and then were used for ion analyses.

For the cations (K^+^, Ca^2+^, Mg^2+^, and Na^+^) and anions (${\text{NO}}_{3}^{-}$, ${\text{PO}}_{4}^{3-}$, and Cl^−^) analysis, 250 mg of dried material was extracted in 50 mL of ultrapure water (Milli-Q, Merck Millipore, Darmstadt, Germany) using a shaking water bath (ShakeTemp SW22, Julabo, Seelbach, Germany) at 80°C for 10 min as described previously by Rouphael et al. (2017b,d). The mixture was centrifuged at 6000 rpm for 10 min (R-10 M, Remi Elektrotechnik Limited, India), then filtered through a 0.20 μm filter paper (Whatman International Ltd., Maidstone, U.K.). The monovalent and bivalent cations were separated by ion chromatography (ICS-3000, Dionex, Sunnyvale, CA, USA) and quantified through an electrical conductivity detector. An IonPac CG12A (4 × 50 mm, Dionex, Corporation) guard column and IonPac CS12A (4 × 250 mm, Dionex, Corporation) analytical column were used for the separation of the four cations, whereas for anions an IonPac AG11-HC guard (4 × 50 mm) column and IonPac AS11-HC analytical column (4 × 250 mm) were used.

### Collection of samples and metabolomic analysis

Two terminal leaflets were sampled from the first fully expanded leaves of two plants per experimental plot at the end of the experiment, and immediately frozen in liquid nitrogen before stored at −80°C for metabolomic analysis. Tissue samples (1.0 g) of four replicates per treatment were extracted in 10+5 mL of 0.1% HCOOH in 80% methanol, using an Ultra-Turrax (Ika T-25, Staufen, Germany), then filtered through a 0.22 μm cellulose membrane disposable filter and finally transferred to an amber vial for analysis. The untargeted metabolite screening was carried out using a 1290 UHPLC liquid chromatography system coupled to a G6550 quadrupole-time-of-flight mass spectrometer, equipped with a JetStream dual Electrospray ionization source (UHPLC-ESI/QTOF-MS) (Agilent Technologies Santa Clara, CA, USA).

The parameters for metabolomic investigations in plant tissues were set out in previous experiments (Pretali et al., 2016). Briefly, chromatographic separation was achieved on an Agilent Zorbax Eclipse-plus column (75 × 2.1 mm i.d., 1.8 μm) using a mobile phase consisting of water (A) and methanol (B), flowing at 220 μL min^−1^ and 35°C. The gradient was initiated with 5% B and increased to 90% B within 35 min, whereas the mass spectrometer was run in positive scan mode (range of 100–1200 m/z) using a nominal mass resolution of 30,000 FWHM. Concerning electrospray conditions, nebulizer pressure was 60 psig, capillary voltage was 4 kV, sheath gas was nitrogen at 10 L min^−1^ (350°C), and drying gas was nitrogen at 10 L min^−1^ (280°C).

Raw data were processed using Profinder B.05 (from Agilent Technologies) for feature initial deconvolution. Compounds identification was carried out using the whole isotopic pattern (i.e., accurate mass, isotope accurate spacing and isotope ratio). Compounds were aligned for both mass and retention time, then annotated using the database PlantCyc 9.5 (Plant Metabolic Network, http://www.plantcyc.org; released November 2014). A filter-by-frequency post-processing was applied retaining only those compounds that were present in 100% of replications within at least one treatment. Therefore, identification was carried out as Level 2 (putatively annotated compounds), according to COSMOS Metabolomics Standards Initiative (http://cosmos-fp7.eu/msi).

### Statistical analysis of experimental data

Experimental data were subjected to two-way analysis of variance (ANOVA) using the SPSS 10 software package. Treatment means within each measured parameter were separated by Duncan's multiple range test performed at a significance level of *P* ≤ 0.05. Principal component analysis (PCA) was also performed using Minitab 16.2.1 statistical software, aimed to extract trends by formulating new variables correlated to the original ones (Lawless and Heymann, 2010; Rouphael et al., 2017d). The PCA outputs included variable loading to each selected component and treatment component scores (Ciarmiello et al., 2015; Rouphael et al., 2017c).

Metabolomics data were formerly elaborated using Agilent Mass Profiler Professional B.12.06 (from Agilent Technologies). Compounds were filtered by abundance (area > 10,000 counts), normalized at the 75th percentile and baselined to the median of control. Unsupervised hierarchical cluster analysis was carried out setting similarity measure as “Euclidean” and “Wards” linkage rule. Fold-change analysis was also carried out, using a cut-off value of 2. Thereafter, the dataset was exported onto SIMCA 13 (Umetrics, Malmo, Sweden), UV-scaled and elaborated for partial least square discriminant analysis (PLS-DA) and Orthogonal Projections to Latent Structures Discriminant Analysis (OPLS-DA) modeling together with unsupervised methods (Worley and Powers, 2013). Hierarchical cluster analysis can be applied in order to reveal differences between classes without supervision, whilst the utilization of class membership in OPLS-DA allows a better separation between classes in score plot hyperspace while effectively separating Y-predictive variation from Y-uncorrelated variation in X. In particular, OPLS-DA allowed separating variation between the groups into predictive and orthogonal (i.e., ascribable to technical and biological variation) components. Outliers were excluded using the distance from the origin in the OPLS-DA model, according to Hotelling's T2 and adopting 95 and 99% confidence limits for suspect and strong outliers respectively. Model overfitting was excluded through cross validation CV-ANOVA (*p* < 0.01) and permutation testing. Model parameters (goodness-of-fit R^2^Y and goodness-of-prediction Q^2^Y) were also produced. Regarding Q^2^Y prediction ability, a value >0.5 was adopted as a threshold to identify acceptable models, according to software recommendation and as set out in literature (Rombouts et al., 2017). Variable importance in projection (VIP analysis) was used to evaluate the importance of metabolites and to select those having the highest discrimination potential (VIP score >1.3). To achieve information on the regulation of biochemical processes related to OMP treatment either under salinity or nonsaline control, a following fold-change analysis was performed for those metabolites highlighted by VIP analysis.

## Results

### Morphological parameters, yield, and root characteristics

**Figure 1:**
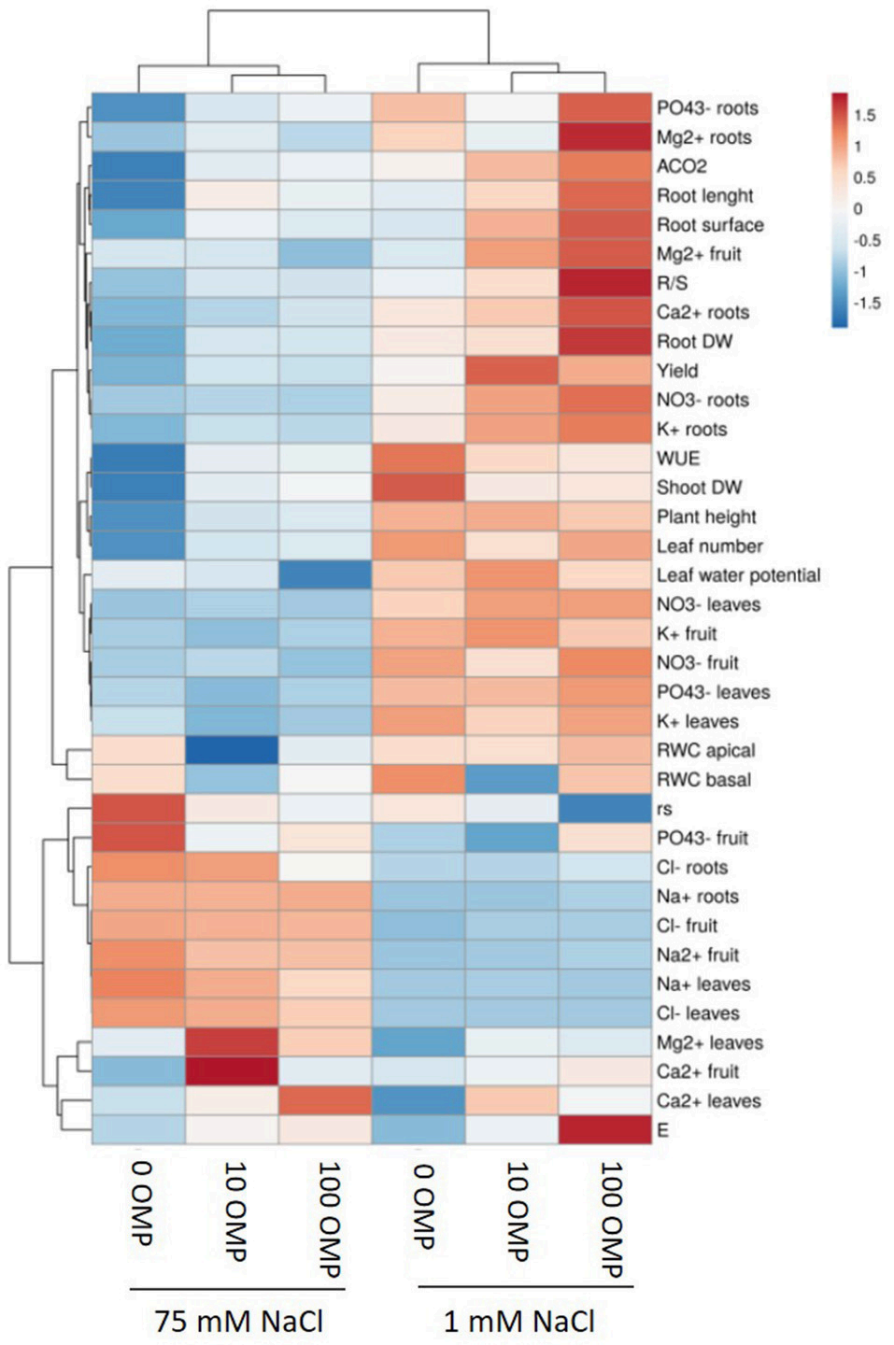
Heat map analysis summarizing the plant responses to NaCl concentration in the nutrient solution and OMP treatments. Results were calculated as Logarithm base 2 (Log2) of untreated and OMP-treated plants under to salinity levels (1 or 75 mM NaCl) and were visualized using a false color scale with red indicating an increase and blue a decrease of plants values compared to values relative to those in control condition. No differences were visualized by white squares.

**Figure 2:**
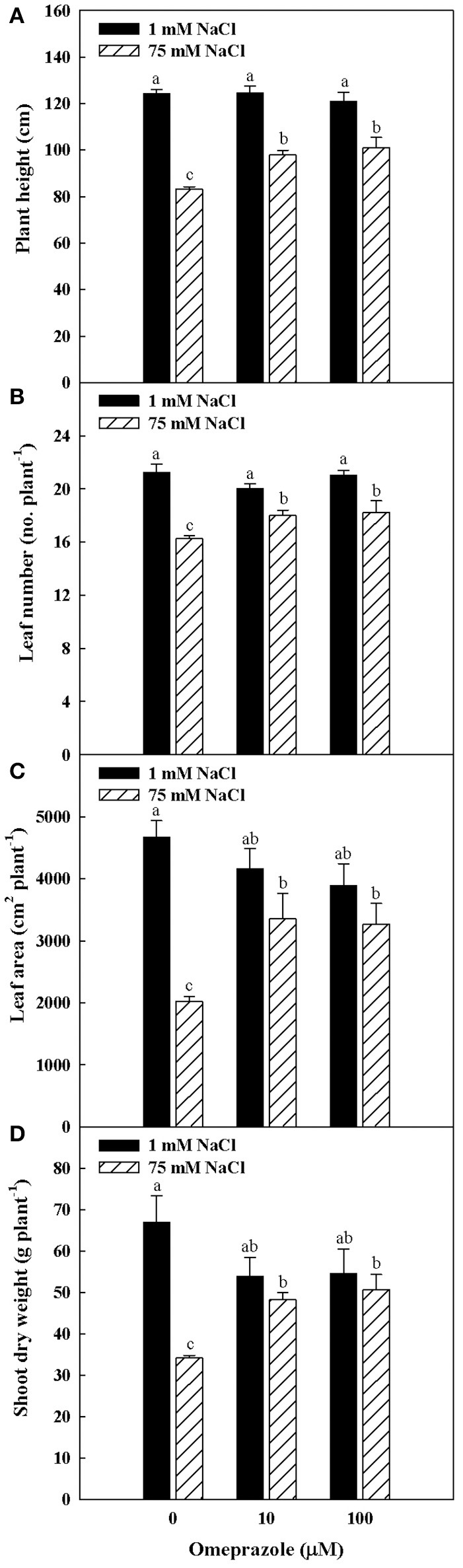
Effects of NaCl concentration in the nutrient solution and omeprazole application on plant height **(A)**, number of leaves per plant **(B)**, total leaf area **(C)**, and shoot dry biomass **(D)** of greenhouse tomato plants. Different letters indicate significant differences according to Duncan's test (*P* = 0.05). The values are the means of four replicate samples. Vertical bars indicate ± SE of means.

Plant height, number of leaves per plant, total leaf area as well as shoot biomass were influenced by salinity and omeprazole (OMP) treatments with significant salinity × OMP interaction. In treated and untreated tomato plants, the plant height number of leaves, leaf area and dry biomass decreased as the salinity level increased, with a more detrimental effect recorded in untreated plants (Figures 1, 2). In fact, increasing NaCl concentration in the nutrient solution from 1 to 75 mM decreased the tomato shoot biomass by 49% in the control treatment, whereas the dry shoot reduction was not significant when 10 μM (−10%) and 100 μM (−7%) of OMP were used, with no significant difference between the two OMP concentrations.

**Figure 3:**
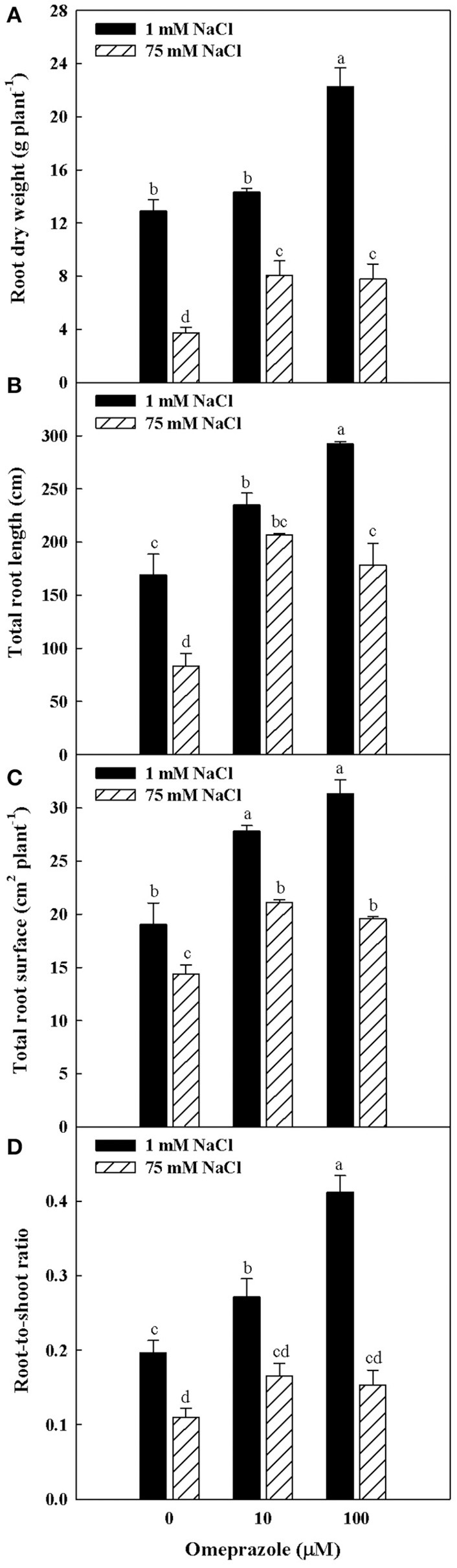
Effects of NaCl concentration in the nutrient solution and omeprazole application on root dry weight **(A)**, total root length **(B)**, total root surface **(C)**, and root-to-shoot ratio **(D)** of greenhouse tomato plants. Different letters indicate significant differences according to Duncan's test (*P* = 0.05). Vertical bars indicate ± SE of means.

Except from the root diameter, which was not affected by either salinity or OMP, root dry weight, total root length and surface as well as the root-to-shoot ratio (R/S) incurred significant salinity × OMP interaction (Figure 1). The root dry weight, total length and surface area were negatively influenced by salt stress treatment (Figure 3). Under nonsaline conditions, the drench application of OMP elicited dose-dependent increases in root dry weight, total length and surface, whereas under saline conditions significant differentiation was observed with respect to the 0 μM control but not between the 10 and 100 μM treatments (Figures 1, 3).

**Figure 4:**
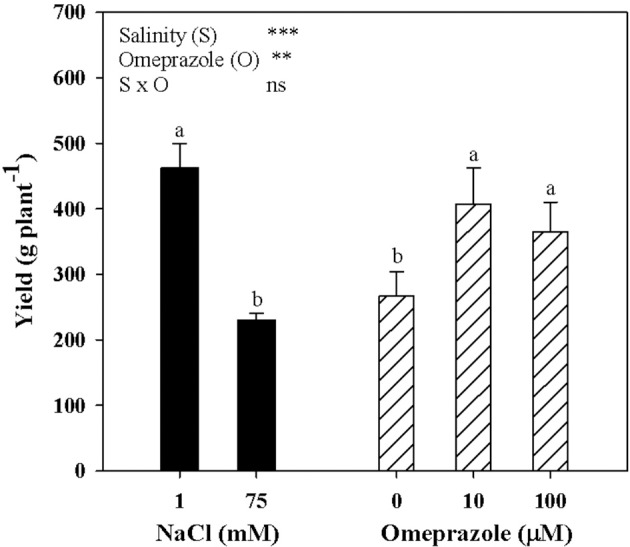
Mean effects of NaCl concentration in the nutrient solution and omeprazole application on yield of greenhouse tomato plants. Different letters indicate significant differences according to Duncan's test (*P* = 0.05). The values are the means of four replicate samples. Vertical bars indicate ± SE of means. ns, ^**^, ^***^ Nonsignificant or significant at *P* ≤ 0.01, and 0.001.

Tomato yield and the mean fruit weight were significantly affected by salinity and OMP treatments with no salinity × OMP interaction. Neither salinity nor OMP treatment had a significant effect on tomato fruit number (data not shown). Irrespective of OMP treatment, fresh tomato yield decreased with increasing salinity in the nutrient solution (Figure 4). Moreover, when averaged over salt-treatment levels, the yield of OMP-treated plants was higher than those of untreated plants by 44.5% (Figure 4).

### Physiological parameters

The net CO_2_ assimilation rate (A_CO2_) and stomatal resistance (r_s_) of tomato plants were significantly affected by salinity and OMP treatments, with no salinity × OMP application interaction; whereas the leaf water potential (Ψl) and WUE were only affected by the salinity treatment (Table 1). Increasing the sodium chloride concentration in the nutrient solution from 1 to 75 mM reduced Ψl, A_CO2_, and WUE by 27, 37, and 34%, respectively, while it increased r_s_ values by 23% (Table 1). Substrate drench application of OMP induced significant increase of A_CO2_ (+48%), with no significant difference between the two OMP concentrations (Table 1). The higher A_CO2_ in OMP-treated tomato plants was accompanied by an increase in E values. Averaged over salinity treatments, OMP application induced lower values of r_s_ in comparison to untreated plants (Table 1). Conversely to the leaf gas exchange parameters, no significant differences between treatments were recorded in RWC of basal leaves (Table 1).

**Table 1:** Analysis of variance and mean comparisons for leaf water potential (Ψ_l_), relative water content (RWC) of apical and basal leaves, net CO_2_ assimilation rate (A_CO2_), stomatal resistance (rs), transpiration rate (E), and water use efficiency (WUE) of tomato plants grown under two salinity levels and treated with omeprazole (OMP) at three rates of application.

**Source of variance**	**Ψ_l_**	**RWC apical**	**RWC basal**	**A_CO2_**	**r_s_**	**E**	**WUE**
	**(MPa)**	**(%)**	**(%)**	**(μmol CO_2_ m^−2^ s^−1^)**	**(m^2^ s^1^ mol^−1^)**	**(mol H_2_O m^−2^ s^−1^)**	**(μmol CO_2_ mol^−1^ H_2_O)**
Salinity (S)	^***^	ns	ns	^***^	^*^	ns	^*^
Omeprazole (OMP)	ns	^*^	ns	^*^	^**^	^*^	ns
S × O	ns	ns	ns	ns	ns	ns	ns
**SALINITY (mM NaCl)**							
1	−1.39a	83.88	91.51	6.22a	16.84b	1.78	3.58a
75	−1.90b	77.33	86.94	3.95b	20.64a	1.69	2.35b
**OMEPRAZOLE (**μ**M)**							
0	−1.58	83.37a	95.57	3.87b	22.06a	1.41b	3.02
10	−1.54	76.36b	81.36	5.46a	18.65ab	1.70ab	2.95
100	−1.81	82.10a	91.81	5.92a	15.52b	2.03a	2.93
**S** × **OMP**							
1 mM NaCl × 0 μM OMP	−1.42	83.29	96.66	5.22	19.86	1.38	4.13
1 mM NaCl × 10 μM OMP	−1.28	83.08	80.14	6.36	17.73	1.66	3.41
1 mM NaCl × 100 μM OMP	−1.46	85.28	93.93	7.09	12.91	2.27	3.16
75 mM NaCl × 0 μM OMP	−1.74	83.44	92.31	2.53	24.25	1.46	1.37
75 mM NaCl × 10 μM OMP	−1.80	69.64	82.57	4.56	19.56	1.73	2.64
75 mM NaCl × 100 μM OMP	−2.15	78.92	88.63	4.75	18.12	1.79	2.70

ns, ^*^, ^**^, ^***^ Nonsignificant or significant at P ≤ 0.05, 0.01, and 0.001, respectively. Different letters within each column indicate significant differences according to Duncan's multiple-range test (P = 0.05).

### Ion content and partitioning

Except for the bivalent cations (Ca^2+^ and Mg^2+^) in leaf tissue, the ${\text{NO}}_{3}^{-}$, ${\text{PO}}_{4}^{3-}$, K^+^ in both leaves and roots as well as Ca^2+^ and Mg^2+^ in roots, were negatively affected by 75 mM NaCl in the nutrient solution (Table 2). Moreover, the concentrations of both toxic elements (Na^+^ and Cl^−^), which accumulated mainly in leaves and to a lesser extent in roots, were significantly influenced by salt stress treatment (Table 2). In OMP untreated plants, the concentrations of Na^+^ and Cl^−^ were 50- and 23-fold higher as the salinity level in the nutrient solution increased (Table 2). The K^+^/Na^+^ ratio, initially equal to 21.4 in leaves and 11.9 in roots, was drastically reduced at 75 mM of NaCl to a value of 0.3 and 0.4, respectively.

**Table 2:** Analysis of variance and mean comparisons for nitrate, phosphate, potassium, calcium, magnesium, sodium and chloride ions in leaves and roots of tomato plants grown under two salinity levels and treated with omeprazole (OMP) at three rates of application.

**Source of variance**	${\text{NO}}_{3}^{-}$ **(mg g**^**−1**^**dw)**		${\text{PO}}_{4}^{3-}$ **(mg g**^**−1**^**dw)**		**K**^**+**^ **(mg g**^**−1**^**dw)**		**Ca**^**2+**^ **(mg g**^**−1**^**dw)**		**Mg**^**2+**^ **(mg g**^**−1**^**dw)**		**Na**^**+**^ **(mg g**^**−1**^**dw)**		**Cl**^**−**^ **(mg g**^**−1**^**dw)**	
	**Leaves**	**Roots**	**Leaves**	**Roots**	**Leaves**	**Roots**	**Leaves**	**Roots**	**Leaves**	**Roots**	**Leaves**	**Roots**	**Leaves**	**Roots**
Salinity (S)	^***^	^***^	^***^	^*^	^***^	^***^	ns	^***^	ns	^***^	^***^	^***^	^***^	^***^
Omeprazole (OMP)	^**^	^**^	ns	ns	ns	^*^	^*^	ns	ns	^*^	ns	ns	^*^	ns
S × O	ns	^*^	ns	ns	ns	ns	ns	ns	ns	^**^	^*^	ns	^*^	ns
**SALINITY (mM NaCl)**														
1	24.33a	26.29a	23.1a	8.81a	39.46a	26.45a	18.99	4.10a	4.80	1.83a	2.82b	1.94b	10.16b	4.19b
75	3.32b	3.24b	16.0b	6.37b	26.42b	9.83b	20.34	2.28b	5.15	1.24b	81.12a	17.89a	173.94a	21.11a
**OMEPRAZOLE (**μ**M)**														
0	11.96b	9.89b	11.3	6.99	34.23	14.04b	16.95b	2.77	4.75	1.47b	48.63	9.84	99.81a	12.83
10	14.90a	16.09a	19.0	7.10	31.27	19.75a	20.73a	3.12	5.16	1.40b	43.04	9.63	94.28ab	12.32
100	14.61a	18.31a	20.0	8.68	33.32	20.63a	21.31a	3.68	5.01	1.73a	34.25	10.27	82.06b	12.81
**S** × **OMP**														
1 mM NaCl × 0 μM OMP	21.19	17.33b	22.8	8.92	40.61	20.76	16.00	3.53	4.62	1.8b	1.9c	1.75	8.29c	3.35
1 mM NaCl × 10 μM OMP	25.84	28.43a	22.7	7.49	37.51	28.11	21.47	3.95	4.91	1.43c	3.67c	1.52	10.89c	3.49
1 mM NaCl × 100 μM OMP	25.96	33.12a	23.9	10.02	40.26	30.48	19.49	4.83	4.86	2.25a	2.9c	2.53	11.29c	5.74
75 mM NaCl × 0 μM OMP	2.73	2.46c	16.4	5.06	27.85	7.32	17.90	2.01	4.88	1.14c	95.35a	17.92	191.33a	22.30
75 mM NaCl × 10 μM OMP	3.96	3.74c	15.4	6.71	25.04	11.39	19.99	2.30	5.41	1.37c	82.41ab	17.73	177.66a	21.15
75 mM NaCl × 100 μM OMP	3.27	3.5c	16.3	7.34	26.37	10.78	23.14	2.53	5.16	1.21c	65.6b	18.01	152.84b	19.88

ns, ^*^, ^**^, ^***^ Nonsignificant or significant at P ≤ 0.05, 0.01, and 0.001, respectively. Different letters within each column indicate significant differences according to Duncan's multiple-range test (P = 0.05).

The OMP treatment, averaged over salt stress levels, affected ${\text{NO}}_{3}^{-}$ and Ca^2+^ concentrations in leaf tissue which were higher by about 23% than in OMP untreated tomato plants (Table 2). Interestingly, under nonsaline conditions the application of 10 and 100 μM of OMP as substrate drench induced a significant increase of ${\text{NO}}_{3}^{-}$ in root tissue (Table 2). Significant OMP × salinity interaction was observed as the OMP treatment effectively reduced the Na+ and Cl− accumulation in leaf tissue under saline (75 mM NaCl) but not under nonsaline (1 mM NaCl) conditions. Under saline conditions the OMP application significantly reduced Na^+^ and Cl^−^ accumulation in leaf tissue in a dose-dependent manner: −14 and −31% Na^+^, and −7 and −20% Cl^−^ in response to 10 and 100 μM OMP, respectively. However, significant reductions in leaf Na^+^ and Cl^−^ concentrations were attained in response to the 100 μM OMP level. Root Na^+^ and Cl^−^ concentrations were lower by 31 and 20%, respectively, when 100 μM OMP was delivered to tomato plants (Table 2, Figure 1).

The fruit mineral composition was significantly affected by salinity and to a lesser extent by the OMP application. Increasing the NaCl concentration in the nutrient solution decreased the concentrations of ${\text{NO}}_{3}^{-}$, K^+^ and Mg^2+^, whereas an opposite trend was observed for Na^+^ and Cl^−^ (Supplementary Table 1). Finally, the highest Mg^2+^ concentration in tomato fruit was observed at 1 mM NaCl combined with the application of 10 and 100 μM OMP (Supplementary Table 1).

### Metabolic profiling of leaves

The salinity × OMP application interaction was also analyzed using an untargeted metabolomics approach based on UHPLC-ESI/QTOF-MS. Overall, this analytical approach allowed annotating 2,019 compounds. The entire list of compounds identified across the samples is provided as Supplementary Table 2, together with annotations and composite MS spectra.

**Figure 5:**
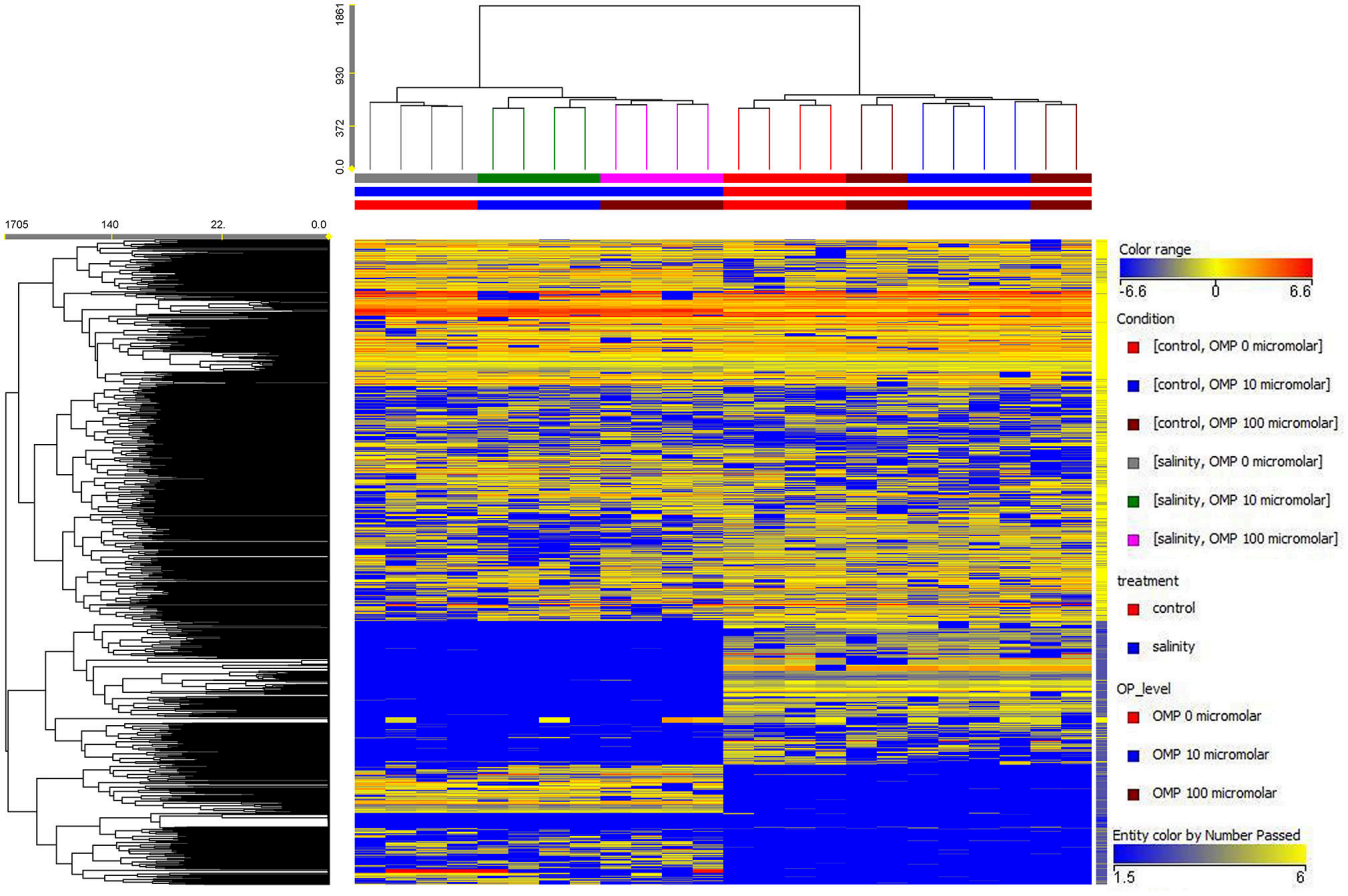
Unsupervised hierarchical cluster analysis of leaf samples from metabolomic profile of tomato plants grown under nonsaline (1 mM NaCl) or saline nutrient solution (75 mM NaCl), following OMP application at three rates (0, 10, or 100 μM). Clustering was carried out on both conditions (treatments, vertical dendrogram) and compounds (metabolites, horizontal dendrogram). Dendrograms were built on the basis of fold-change based heat map (similarity: Euclidean, linkage rule: Ward).

**Figure 6:**
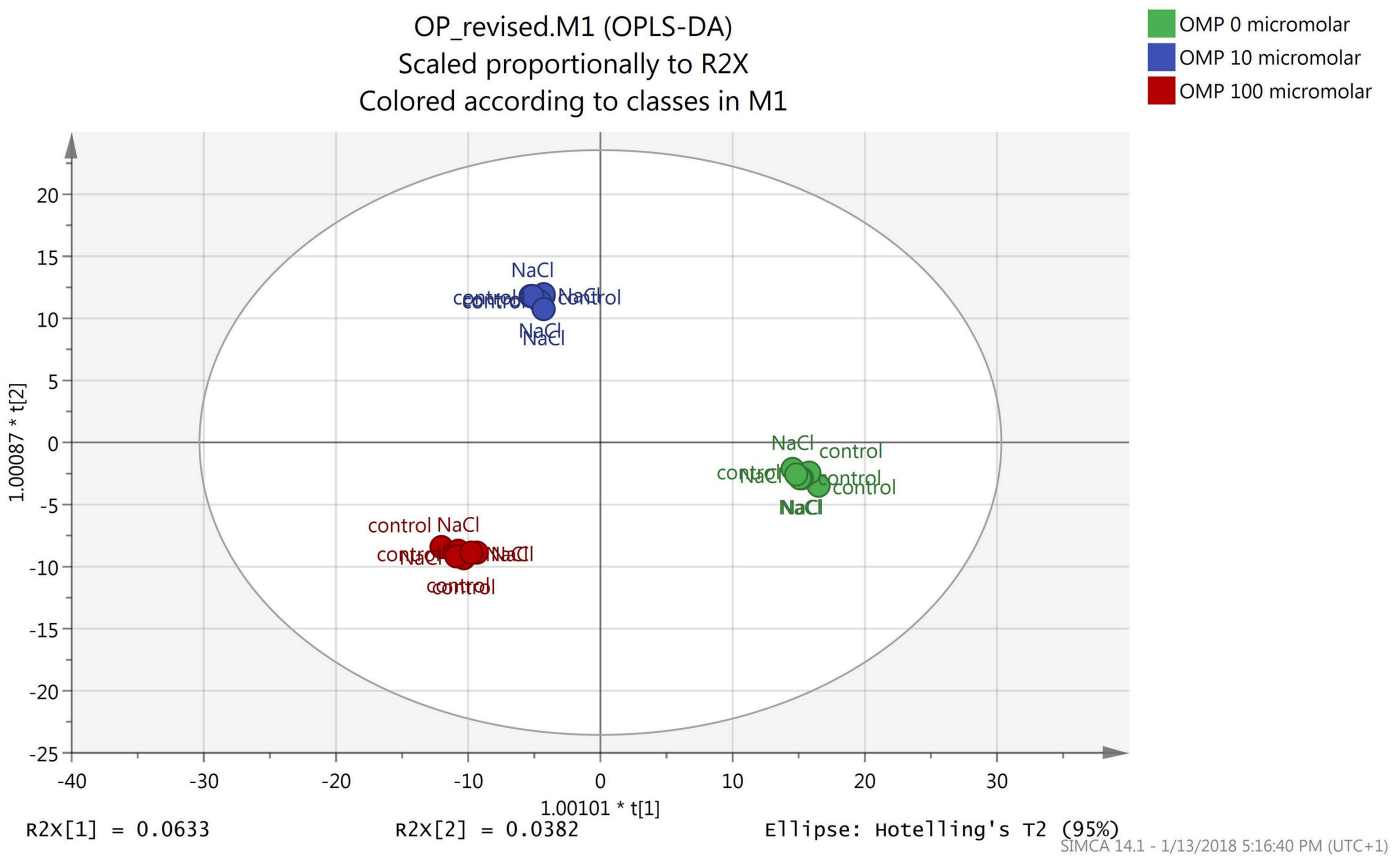
Orthogonal Projections to Latent Structures Discriminant Analysis (OPLS-DA) on tomato leaves metabolome from plants grown under nonsaline (1 mM NaCl) or saline nutrient solution (75 mM NaCl), following OMP application at three rates (0, 10, or 100 μM). Individual replications are given in the class prediction model score plot.

Both the non-averaged unsupervised hierarchical cluster analysis and the supervised orthogonal projection to latent structures discriminant analysis (OPLS-DA) multivariate statistical approaches (see Materials and Methods section) allowed differentiating between treatments (Figures 5 and 6), suggesting that the metabolic profiles were affected by the treatments. In particular, when looking at the heat-map based on the fold-change analysis (Figure 5), two main clusters could be identified, representing 1 and 75 mM NaCl respectively. These findings indicated that salinity was the main clustering factor. Nonetheless, OMP treated plants could be discriminated from those without OMP, and a dose-dependent response was also observed under salt stress conditions (i.e., 75 mM NaCl). Indeed, three separated sub-clusters could be defined under salinity, whereas OMP treated plants clustered together under 1 mM NaCl conditions. The following OPLS-DA supervised multivariate analysis provided an output that was consistent with hierarchical clustering, suggesting that salinity was the principal factor followed by OMP concentration (Figure 6). To better point out the response related to OMP itself rather than its specific biochemical role in promoting salt stress tolerance, two different OPLS-DA models were built, under 1 and 75 mM NaCl, respectively (Supplementary Figure 1). Both models fitting parameters were more than adequate, being goodness-of-fit R2Y = 0.99 under both salinity conditions and goodness-of-prediction Q2Y 0.59 and 0.73 for 1 and 75 mM NaCl, respectively. Both models provided 100% accuracy in class prediction (Fischer's probability: 0.0002), whereas cross validation CV-ANOVA (*p* < 0.01) and permutation testing excluded model overfitting. No outlier replicates could be identified using Hotelling's T2 under 95 and 99% confidence limits for suspect and strong outliers, respectively.

Given the adequate fitting of OPLS-DA models, a subsequent investigation was done aiming to identify the compounds differences could be attributed to. With this purpose, the investigation of the most discriminant compounds in the OPLS-DA model (i.e., variables of importance in projection—VIP analysis) was carried out. Table 3 reports the metabolites identified (i.e., >1.3) by VIP analysis, together with individual scores and their standard error, as well as Log fold-change values and regulation. Overall, 84 compounds were identified as those variables mostly contributing to class discrimination in OPLS-DA. Discriminating compounds were grouped in functional classes; hormones, membrane lipids, terpenes, and alkaloids were the most represented classes. Among hormones, compounds related to almost all classes could be discerned. In more detail, the brassinosteroid brassinolide, auxin inactivation compounds (oxindole-3-acetyl-aspartate-N-beta-glucosyl-beta-1,4-glucose; 2-oxindole-3-acetyl-hexose; indole-3-acetyl-tryptophan), inactive forms of gibberellins (A34, A98, A51-catabolite), a precursor and a catabolite of abscisic acid (abscisic aldehyde and dihydroxyphaseic acid respectively), methyl jasmonate and a cytokinin (trans-zeatin riboside triphosphate) were identified. Among lipids, several membrane lipids (glyco- and phospholipids) were identified in VIP analysis, together with cutin biosynthetic intermediates [9,10-epoxystearate and (9R,10S)-dihydroxystearate]. Sesquiterpene lactones were also among discriminating compounds, including lubimin-related sesquiterpenoid phytoalexins (3-hydroxylubimin and 2-dehydrolubimin), parthenolide, two costunolide-related compounds [3-beta-hydroxycostunolide and germacra-1(10),4,11(13)-trien-12-oate] as well as zealexins A1 and A3. Furthermore, several alkaloids and phenolics were outlined as OPLS-DA discriminants; among the seconds, tetramethylquercetagetin, conjugated cyanidins, and hydroxycinnamics were the most represented. However, ajmaline and sarpagine, lupanine, and cinchona alkaloids were the most common. Carotenoids (mainly ascribable to xanthins), polyamines and their conjugates, pteridine as well as porphyrin biosynthetic precursors were also selected among discriminating compounds. Among amino acids, asparagine, lysine, saccharopine (a lysine degradant), and cystathionine (involved in cysteine/homocysteine interconversion) were pointed out. Finally, some other compounds could be recognized as differential, including L-dopachrome (intermediate in eumelanin biosynthesis), 6,7-dimethyl-8-(1-D-ribityl) lumazine (flavin biosynthesis), a plastoquinone, an acetyl-hexosamine and two glucosinolate-related compounds (9-methylthiononylhydroximoyl-glutathione and 7-methylthioheptyldesulfoglucosinolate).

**Table 3:** Leaf metabolites discriminating tomato plants under two salinity levels and treated with omeprazole (OMP) at three rates of application.

**Compound**	**OPLS-DA VIP**		**[OMP 100** μ**M] vs. [OMP control], 1 mM NaCl**		**[OMP 100** μ**M] vs. [OMP control], 75 mM NaCl**	
	**Score**	**Standard error**	**Log fold-change**	**Regulation**	**Log fold-change**	**Regulation**
**AMINO ACIDS**						
L-asparagine	1.33	0.89	−4.13	Down	−3.18	Down
L-cystathionine	1.33	1.31	0.00	Down	−1.50	Down
L-lysine	1.39	0.73	0.52	Up		
L-saccharopine	1.31	0.45	0.00	Down		
**HORMONES**						
brassinolide	1.38	0.36	−0.37	Down	−2.98	Down
oxindole-3-acetyl-aspartate-N-beta-glucosyl-beta-1,4-glucose	1.30	0.39	−6.66	Down	−1.75	Down
a 2-oxindole-3-acetyl-hexose	1.64	0.54	−4.40	Down		
indole-3-acetyl-tryptophan	1.56	0.43	6.72	Up		
gibberellin Asub34/sub	1.47	0.17	20.34	Up		
gibberellin Asub98/sub	1.39	0.85	−5.29	Down	−1.35	Down
gibberellin Asub51/sub-catabolite	1.32	0.67	−9.51	Down		
trans-zeatin ribosidetriphosphate	1.38	0.96	0.00	Down	−3.92	Down
(+)-cis-abscisic aldehyde	1.38	1.00	9.30	Up		
dihydroxyphaseic acid	1.36	0.86	5.48	Up	−0.03	Down
methyl jasmonate	1.36	1.08	0.59	Up	−0.01	Down
**LIPIDS**						
9,10-epoxystearate	1.35	0.65	0.37	Up		
(9R,10S)-dihydroxystearate	1.34	0.66	0.45	Up		
1-18:0-2-18:1-phosphatidylethanolamine	1.43	0.79	1.40	Up		
1-16:0-2-18:3-diacylglycerol-trimethylhomoserine	1.39	0.93	4.94	Up	−1.56	Down
1-18:3-2-16:3-monogalactosyldiacylglycerol	1.31	0.96	0.01	Up	−0.23	Down
1-18:1-2-16:1-monogalactosyldiacylglycerol	1.39	0.57	10.83	Up	−6.62	Down
1-18:1-2-16:0-monogalactosyldiacylglycerol	1.36	1.23	9.91	Up	−0.14	Down
1-18:2-2-18:2-monogalactosyldiacylglycerol	1.31	0.90	−0.21	Down	−0.38	Down
1-18:1-2-16:0-phosphatidylglycerol	1.38	0.91	5.20	Up	−2.57	Down
1,2-dipalmitoyl-phosphatidylcholine	1.37	0.40	−5.29	Down	−3.94	Down
1-18:1-2-18:3-phosphatidylcholine	1.43	0.45	−0.15	Down	−2.57	Down
1-18:3-2-18:2-phosphatidylcholine	1.38	0.58	0.11	Up	−0.19	Down
1-18:1-2-18:1-sn-glycerol-3-phosphocholine	1.37	1.06	0.09	Up	−0.60	
1-18:3-2-18:3-phosphatidylcholine	1.35	0.65	−0.13	Down	−2.41	Down
1-18:3-2-18:1-phosphatidylcholine	1.32	0.68	−0.15	Down	−0.60	Down
linolenate	1.34	0.74	6.84	Up		
(9S,10S)-9,10-dihydroxyoctadecanoate	1.34	0.66	0.26	Up		
4-alpha-carboxy-5-alpha-cholesta-8,24-dien-3-beta-ol	1.31	0.93	18.25	Up	−4.50	Down
**SESQUITERPENE LACTONES**						
zealexin A1	1.42	0.71	0.05	Up	−1.65	Down
zealexin A3	1.34	0.87	0.44	Up		
parthenolide	1.38	1.00	9.30	Up		
germacra-1(10),4,11(13)-trien-12-oate	1.42	0.71	−5.10	Down	−1.65	Down
3-beta-hydroxycostunolide	1.38	1.00	9.30	Up		
3-hydroxylubimin	1.50	0.55	4.21	Up		Down
2-dehydrolubimin	1.33	0.78	−4.83	Down		
**ALKALOIDS**						
10-deoxysarpagine	1.38	0.80	−5.41	Down	−1.44	Down
vellosimine	1.36	0.37	−0.20	Down		
17-O-acetylajmaline	1.35	1.03	0.08	Up		
1,3,7,9-tetramethylurate	1.39	1.15	−4.22	Down	−0.08	Down
(S)-n-methylcanadine	1.40	1.05	5.16	Up	−0.08	Down
quinidinone	1.40	0.62	0.42	Up	−5.61	Down
cinchoninone	1.36	0.37	−4.84	Down		
lupanine	1.34	0.60	−0.02	Down		
17-oxosparteine	1.34	0.60	−0.02	Down		
**PHENOLICS**						
glyceollin I/II	1.38	1.06	0.00	Down		
maysin	1.35	0.56	−3.19	Down		
1-naphthol glucoside	1.38	0.87	0.54	Up		
tetramethylmyricetin/tetramethylquercetagetin	1.32	0.67	0.14	Up		
2′-hydroxy 3,6,7,4′-tetramethylquercetagetin	1.32	0.58	0.53	Up	−0.49	Down
cyanidin 3-O-glucoside-7-O-(6-O-(p-hydroxybenzoyl)-glucoside)	1.31	0.65	4.88	Up	−0.07	Down
cyanidin 3-O-glucoside-7-O-(6-O-(4-O(6-O-(p-hydroxybenzoyl)-glucosyl)-oxybenzoyl)-glucoside)	1.49	0.65	−2.81	Down		
cinnamaldehyde	1.43	0.63	14.91	Up		
beta-D-glucosyl-2-hydroxycinnamate	1.40	0.68	0.48	Up	−0.62	Down
**PORPHYRIN**						
protoporphyrin IX	1.38	0.35	−8.53	Down		
uroporphyrinogen-III	1.41	0.94	−0.11	Down		
**CAROTENOIDS**						
3,4,3′,4′-tetradehydroisozeaxanthin	1.41	0.55	6.19	Up		
capsanthin	1.34	0.86	0.12	Up	−0.14	Down
sulcatone	1.31	0.58	0.00	Down		
caloxanthin	1.34	0.86	0.12	Up	−0.14	Down
5,6-epoxy-3-hydroxy-9-apo-beta;-caroten-9-one	1.36	1.08	0.42	Up	−0.01	Down
zeinoxanthin	1.43	1.05	0.24	Up	−0.08	Down
**POLYAMINES AND CONJUGATES**						
dihydroxyferuloyl-sinapoyl spermidine	1.38	0.54	0.00	Down	−3.72	Down
acetylspermidine	1.39	0.46				
tyramine	1.69	0.45	0.00	Up	−7.96	Down
cinnamoyltyramine	1.31	0.88	−4.92	Down		
**PTERIDINE**						
a 5,6,7,8-tetrahydropteridine	1.35	0.35	5.53	Up		
6-hydroxymethyl-7,8-dihydropterin	1.34	1.25	0.21	Up	−2.88	Down
tetrahydropteroyl-&alpha;-glutamylglutamate	1.33	0.80	0.03	Up	−5.91	Down
**OTHERS**						
L-dopachrome	1.33	0.66	−0.08	Down	−1.06	Down
6,7-dimethyl-8-(1-D-ribityl)lumazine	1.33	0.97	0.00	Down	−1.73	Down
(R)-pantoate	1.43	0.47	0.54	Up		
dehydroascorbate (bicyclicform)	1.38	0.49			−0.31	Down
a plastoquinone	1.44	0.82	0.09	Up		
an N-acetyl-D-hexosamine	1.40	0.55	−0.09	Down	−0.82	Down
9-methylthiononylhydroximoyl-glutathione	1.32	0.70	15.32	Up	−4.79	Down
7-methylthioheptyldesulfoglucosinolate	1.31	0.88	5.87	Up		

*Compounds were gained through UHPLC-ESI/QTOF-MS metabolomics and selected by OPLS-DA discriminant analysis followed by VIP (Variables of Importance in Projection) analysis. Compounds are grouped in functional classes and provided together with VIP score, VIP score standard error, as well as fold-change analysis*.

### Principal component analysis

**Figure 7:**
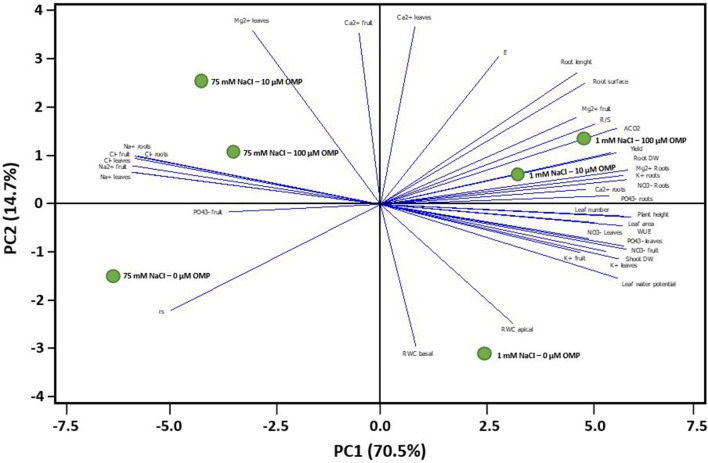
Principal component loading plot and scores of principal component analysis (PCA) of morphological, physiological traits and ion contents of greenhouse tomato grown under nonsaline (1 mM NaCl) or saline nutrient solution (75 mM NaCl), following OMP application at three rates (0, 10, or 100 μM).

To obtain a broad overview on the morphological and physiological changes of greenhouse tomato plants in response to OMP application under both saline and nonsaline conditions, the PCA was carried out. The first two principal components (PCs) were related with Eigen values > 1 and explained 85.2% of the total variance with PC1 and PC2 accounting for 70.5 and 14.7%, respectively (Figure 7). PC1 was positively correlated to nitrate and potassium concentration in leaf tissues, plant height, net photosynthetic rate, yield as well as leaf number and area. PC1 was also negatively correlated to both toxic ions as well as to r_s_. Moreover, PC2 was positively correlated to Ca^2+^ and Mg^2+^ in leaves, transpiration and root length and root surface, and negatively correlated to RWC basal and apical, r_s_ and K^+^ in leaves and fruit. Furthermore, the score plot of the PCA clearly divided the two nutrient solutions (1 and 75 mM NaCl) along PC1 with nonsaline treatment concentrating most of the plant growth parameters, yield and ${\text{PO}}_{4}^{3-}$, K^+^, Ca^2+^, Mg^2+^ in roots and physiological parameters, whereas the saline treatment stands out for toxic ions (Na^+^ and Cl^−^) (Figure 7). The OMP applications were clustered in respect to PC2, with both 10 and 100 μM OMP on the positive side of the PC2 that is characterized by improved physiological status, root characteristics, photosynthetic performance, R/S and yield (at 1 mM NaCl) and higher leaf Mg^2+^ as well as lower Na^+^ and Cl^−^ concentrations in both leaves and roots (at 75 mM NaCl; Figure 7).

## Discussion

### Implications of omeprazole for morphological and physiological parameters

Vegetable crops respond to excessive sodium chloride concentration in soil or irrigation water with growth inhibition and yield reduction (Tester and Davenport, 2003), and the severity of crop production losses may fluctuate in relation to several interconnected variables such as cultural environment, genetic material (species and/or cultivars) as well as the concentration, time of exposure, and type of salts (Colla et al., 2010). The significant depression of plant growth parameters (plant height, leaf number, leaf area, biomass production) as well as yield reduction with increasing NaCl in the nutrient solution has been reported previously in several greenhouse experiments on potted leafy and fruit vegetables, including tomato (Colla et al., 2006; Savvas et al., 2011; Rouphael et al., 2012, 2016, 2017b; Lucini et al., 2016). Furthermore, high concentration of NaCl in the nutrient solution will induce a broad range of biochemical, physiological, anatomical, and metabolic changes such as impairment of root activity, nutrient imbalance, chlorophyll degradation and decrease of the net photosynthetic rate (Munns, 2002; Munns and Tester, 2008), as observed in the present experiment on tomato plants supplied with 75 mM NaCl. Significant decrease in morphological and physiological traits in NaCl-treated tomato plants occurred; and that effect varied in relation to the OMP application. However, the positive effects of OMP, for example on WUE, were found at the end of the salinity treatment, suggesting a time-dependent action of OMP. It is probable that the WUE improved under salinity thanks to another mechanism adopted by plants to minimize water loss even at low r_s_, that is leaf area restriction, a feature shown by both halophytes and non–halophytes under high salinity (Maas and Nieman, 1978).

The expected stimulator effect of OMP, a benzimidazole inhibitor of animal proton pumps, on plant growth parameters (i.e., plant height, leaf number and area, shoot dry biomass), previously reported in Van Oosten et al. (2017), was not evidenced in the absence of salt stress. However, other morphological and physiological parameters were increased by OMP application independently of salinity, in particular root dry weight, root length and surface, R/S, transpiration and photosynthetic net rate. An explanation for this different response, observed against the former study which was performed in the absence of stress, could be attributed to the different growing conditions (hydroponic vs. substrate experiment), variation between the tomato cultivars employed (determinate type “M82” vs. indeterminate type “Seny”) and not least the length of the growth cycle (14 vs. 65 days) as the former study was terminated before the plants reached their reproductive stage. On the other hand, the detrimental impact of NaCl was clearly mitigated when 10 and 100 μM of OMP were applied to tomato plants by substrate drench. The improvement of plant growth parameters induced by OMP application could be associated with the stimulation of the root system architecture (increased root dry biomass, total root surface, and length), which may improve nutrient use efficiency and total biomass production. The application of OMP may have also triggered a signal transduction pathway mediated by endogenous phytohormone (i.e., elicitation of root auxin synthesis), which resulted in significant increase of root length and density, thus inducing a “*nutrient acquisition response”* that favored nutrient uptake and translocation.

Omeprazole application could be also responsible for the inhibition of swelling-dependent chloride channels (Schmarda et al., 2000). The presence of non-isosmotic conditions can alter intracellular and extracellular osmolality, generating a passive flow of water thus causing cell swelling or shrinkage (Sardini et al., 2003). In plants, shrinkage is much more dangerous than swelling that is counteracted by vacuolar and cell wall action. When a cell swells, it attempts to restore its original volume by activating channels or transporters in order to release appropriate osmolytes, typically K^+^, Cl^−^ and organic osmolytes. If the osmolyte and/or ion efflux is blocked (OMP effect), it is possible that root cells undergo an enlargement of cell volume thus having a positive effect on root characteristics (total root length and surface area).

Another putative mechanism supporting the inductive role of OMP in stress tolerance is the higher CO_2_ assimilation rate, through: (i) better osmotic adjustment, (ii) improved balance between the uptake and loss of water, (iii) higher efficiency in absorbing macro and microelements from the substrate, thus boosting tomato performance. In addition, the application of OMP may improve photosynthesis by reducing the stomatal resistance, as observed in the current experiment as well as by Van Oosten et al. (2017).

### Implications of omeprazole for ion homeostasis

The maintenance of ion homeostasis, in which salt overly sensitive (SOS) pathway plays a key role, is a major adaptation strategy against salinity, highly implicated in plant salt tolerance (Soni et al., 2013). In the present study, the high concentration of Na^+^ and Cl^−^ in the nutrient solution depressed cation and anion uptake, translocation and accumulation based on the strong decrease of ${\text{NO}}_{3}^{-}$, ${\text{PO}}_{4}^{-}$, K^+^ (in both leaf and root tissues) and Ca^2+^ and Mg^2+^ (in roots) as previously reported by Grattan and Grieve (1999) on a wide range of horticultural commodities. It is well established that Cl^−^ competition for ${\text{NO}}_{3}^{-}$ transporter proteins affects ${\text{NO}}_{3}^{-}$ uptake and transport and reduces the loading of ${\text{NO}}_{3}^{-}$ into the root xylem (Carillo et al., 2005, and references therein). Moreover, since ${\text{NO}}_{3}^{-}$ is necessary to induce nitrate reductase (NR), the first key enzyme of the nitrogen assimilation process, decrease of ${\text{NO}}_{3}^{-}$ flux from roots under salinity stress severely affects NR and nitrogen assimilation in leaves (Campbell, 1999). High concentration of Na^+^, in turn, impairs not only K^+^ translocation from root to shoot but also its uptake by plasma membrane transport (Gao et al., 2016). Moreover, Na^+^ can depolarize and damage the plasma membrane favoring K^+^ leakage, further decreasing the cytosolic K^+^ content (Wang et al., 2013). When Na^+^ concentration is much higher than that of K^+^, it can substitute K^+^ in key enzymatic reactions and damage metabolic pathways in cytosolic compartments. Therefore, plants are less sensitive to the absolute amount of Na^+^ than to K^+^/Na^+^ ratio (Shabala and Cuin, 2008; Cuin et al., 2009). However, salt tolerant tomato plants were able to retain higher Na^+^ and Cl^−^ levels in leaves than in roots, suggesting the presence of an active inclusion mechanism in plants as a trait of salt tolerance (Läuchli and Epstein, 1990; Rodriguez et al., 2005). In fact, taking into account the high genotypic diversity of tomato plants with respect to ion homeostasis, the more tolerant species/accessions are able to accumulate higher amounts of salts in shoots (leaves and stems), while those more sensitive accumulate salts principally in roots (Cuartero and Fernández-Mu-oz, 1998). It has been ascertained that tomato roots can sense and control the Na^+^ concentration reaching aerial parts depending on the intensity of the stress, probably thanks to the SOS pathway (Olías et al., 2009).

Accordingly, an intriguing current result was that OMP treatment decreased Na^+^ and Cl^−^ concentration in leaves, especially the 100 μM application, and increased leaf Ca^2+^ concentration. However, K^+^/Na^+^ ratio did not significantly increase in leaves but also in the roots of OMP treated plants where K^+^ slightly increased. These findings suggest the induction of a salt-tolerance mechanism other than vacuolar sequestration of Na^+^, which is a common means for decreasing Na^+^ concentration in the cytoplasm, thereby contributing to the osmotic adjustment while maintaining water absorption under salt stress conditions (Silva and Gerós, 2009). Mitochondria and plastids can also sequester some Na^+^ contributing to its compartmentalization (Conde et al., 2011). At the same time, cytosolic K^+^ concentration can be maintained at a constant level or, thanks to the K^+^ stored in the vacuole, even increased, osmoregulating the cell and avoiding the impairment of plant metabolism under salinity.

### Implications of omeprazole for the metabolomic profile of tomato leaves

The metabolomic profile of tomato leaves was clearly affected by the OMP treatment as highlighted by both unsupervised and supervised multivariate statistics. Hierarchical cluster analysis, i.e., the unsupervised chemometric approach, evidenced two distinct clusters, comprising NaCl saline stress and nonsaline control. Looking at sub-clusters within main clusters, the effect of OMP was still evident, resulting in a mixed cluster in control (comprising both 10 and 100 μM OMP) separate from 0 μM OMP. However, three distinct sub-clusters could be evidenced in the main salinity cluster representing 0, 10, and 100 μM OMP. The former clustering, therefore, evidenced that OMP treatment had an impact on tomato leaf metabolomic profile even under nonsaline conditions. This effect became dose-related when 75 mM NaCl was applied. On these premises, it can be postulated that the effect of OMP far exceeded the osmoregulation of ions. This is coherent with the fact that OMP is known to interfere with P-Type IIC ATPases, a large family of ATP-driven transporters (Shin et al., 2009) that have not been reported in *planta* (Van Oosten et al., 2017). As a further confirmation, Na concentration in salinized roots was unrelated to the OMP treatment. Interestingly, looking at plants grown in 75 mM NaCl, Na^+^ concentration in leaves was reduced by OMP in a dose-dependent manner but the K^+^/Na^+^ ratio was almost unaffected. These findings support the fact that a complex metabolic response might be involved, potentially including hormonal network balance and compounds trafficking.

To investigate further the effect of OMP on tomato leaf metabolome, a supervised tool was carried out. It is reported that OPLS-DA is a powerful supervised approach in metabolomics (Worley and Powers, 2013). Indeed, the utilization of class membership in OPLS-DA allows a better separation between classes in score plot hyperspace, while effectively separating Y-predictive variation from Y-uncorrelated variation in X. The VIP score, being calculated as a weighted sum of the squared correlations between the OPLS-DA components and the original variables, is next able to summarize the contribution a variable provides to the model. The excellent OPLS-DA model parameters achieved starting from UHPLC-ESI/QTOF-MS profiles, suggest that differences were actually represented within our dataset.

Hormone compounds were among the most represented in VIP analysis. A complex fine-tuning of plant hormone profiles is occurring under salt stress conditions, as recently reviewed (Ryu and Cho, 2015). Abscisic acid (ABA) is a key enzyme in regulating the response to saline stress since its increase induces stomatal closure, accumulation of osmolytes and growth defects thus ensuring plant survival under salinity. Indeed, ABA precursors accumulated in OMP-treated tomato under 1 mM NaCl, suggesting that the treatment might trigger an improved tolerance to salinity. Coherently, a decrease was observed for both auxins (catabolites decreased and an inactive conjugate form increased under 1 mM NaCl) and a cytokinin (a zeatin-riboside derivative in 75 mM NaCl). Auxins are known to cause hypersensitivity to salt stress, likely because they interfere with the salt-mediated remodeling of root architecture (Ryu and Cho, 2015 and references therein). Analogously, cytokinins have a negative role in response to salinity, as their receptors modulate environmental signals and because of their ABA-antagonistic activity (Ryu and Cho, 2015 and references therein). Finally, also a decrease in gibberellins (GA) can trigger salt tolerance, as GA-deficient mutants exhibited enhanced salt stress response (Ryu and Cho, 2015). Notably, GA catabolites were found among discriminant metabolites in our experiments. It must be also pointed out that a complex network of cross-talking enzymes might be considered. As an example, it is known that auxin regulation of GA biosynthesis has a key role in regulating growth between different organs/tissues, and that this aspect relates to survival under salinity conditions (Yamaguchi, 2008). Ethylene is a further hormone known to play a central role in abiotic stress response. Our analytical approach could not detect ethylene, as this is a volatile small metabolite. The brassinosteoid brassinolide was also involved in OMP-related response. Brassinosteroids are involved in plant stress response through cross-talking with ABA, and are supposed to have a positive role in stress tolerance via modulation of stomatal conductance (Ryu and Cho, 2015 and references therein). Unexpectedly, the current trend was not consistent with previous findings, as brassinolide down accumulated in 75 mM NaCl treated tomato plants. Nonetheless, the decrease in L-cystathionine (involved in *de novo* synthesis of ethylene precursor methionine) was found down accumulated in OMP-treated tomato leaves under 75 mM NaCl. Although the changes in phytohormonal profile induced by OMP deserve further investigation, the above-reported information clearly suggests that OMP treatment significantly altered hormonal balance in tomato. Looking at the changes in growth and physiological parameters, these OMP-induced alterations might have contributed toward the increase in salt stress tolerance we observed.

Besides hormonal imbalance, lipids were also involved in the response to OMP treatment. Interestingly, two cutin-related compounds were up accumulated in leaves treated with OMP. Cuticular lipids are reported to be induced by NaCl and drought, with the aim of limiting water losses thanks to their ability to postpone the onset of cellular dehydration (Kosma et al., 2009). Several membrane lipids, mainly glycosylated lipids or phospholipids, were also involved in stress response. The plasma membrane H^+^ ATPase pumps play essential roles in signal transduction, cell expansion, stomatal opening, and salt stress response (Sun et al., 2010a). These pumps counteract salt activated K^+^ efflux, which is mediated by depolarization activated channels and accelerate H_2_O_2_ production via NADPH oxidases (Zhang et al., 2017), thus triggering Ca^2+^ influx (Sun et al., 2010b). Subsequently, elevated Ca^2+^ levels activate the SOS signaling pathway (Zhang et al., 2017). SOS pathway alters, among others, the cytoskeleton, root architecture and mineral partitioning (Ji et al., 2013). The changes observed in membrane lipids might be the result of these membrane related processes occurring under salinity. Analogously, the accumulation in phenolics and carotenoids could be related to the increased H_2_O_2_ production, with the aim of strengthening the antioxidant capacity of leaves under salt stress. Coherently, the oxidized form of ascorbic acid was found to be down accumulated in OMP treated plants under 75 mM NaCl.

Also terpenes and alkaloids are compounds that can be triggered by environmental stress, and by NaCl salinity in particular (Chadwick et al., 2013; Lucini et al., 2015); among terpenes, sesquiterpenoids are reported to possess antioxidant capacity (Chadwick et al., 2013), and therefore might be also implicated in the aforementioned response to oxidative stress at the membrane level.

Spermidine and tyramine polyamine conjugates were also altered by OMP treatment. These compounds can be acylated for regulatory purposes likely altering their biological functionality; they are reported to be involved in a wide range of plant developmental processes such as cell division, flowering, and responses to environmental stress (Luo et al., 2009). Although their specific role in OMP response is still unclear, their recruitment was highlighted in the present results, consistently with previous findings on abiotic stress response (Shi and Chan, 2014; Rouphael et al., 2016). The involvement of pteridine, needed for folate biosynthesis, is also not surprising. Indeed, plant metabolism involves a wide range of interconversion and donation of one-carbon (C1) units, through reactions where folates are essential cofactors. Folates participate moreover in the THF-mediated glycine-serine conversion in photorespiration.

Overall, a very articulated and complex metabolic response was observed in response to OMP treatment, involving hormonal balance and cross-talking, membrane processes and oxidative stress there occurring under salinity, as well as a range of other stress elicited chemical compounds. These processes differ from the classical mechanisms through which plants increase tolerance to salinity. Typically, salt-resistant plants possess an improved capacity for H^+^ pumping activity, which enables salinized cells to retain K^+^/Na^+^ homeostasis and avoid ionic toxicity. According to our results, the processes related to OMP treatment transcend typical ion homeostasis. Although a single well-defined specific mechanism could not be outlined, a hormone-like activity has been postulated. On this basis, the mechanism(s) through which OMP affects tolerance to NaCl salt stress involve different biochemical/metabolic responses. Taken together, these OMP-related changes finally end in an improved capacity of counteracting the detrimental processes triggered by salinity.

## Conclusions

Under climate change scenario, the pressure of abiotic stressors and in particular salinity on vegetable productivity is expected to further challenge food security in the coming decades. Thus, it is important to explore the potential role of small bioactive molecules resourced from human/animal research in increasing vegetable plant tolerance to conditions of salinity. The metabolic profile of plants was found significantly affected by OMP treatment, and dose-dependent changes in key metabolites were identified under 75 mM NaCl salt stress conditions. OMP was not strictly involved in homeostasis of ions, even though it was able to decrease leaf Na^+^ and Cl^−^ concentration under salinity stress. This is in agreement with the fact that plants do not possess P-Type IIC ATPases, i.e., the known target of OMP. However, this small bioactive molecule appeared to be involved in a signal transduction pathway regulating endogenous hormones responsible for the increase of morphological root parameters, and consequently for the “*nutrient acquisition response.”* Hormonal network was significantly affected by OMP, eliciting increase in ABA, decrease in auxins and cytokinin, as well as a tendency in GA down accumulation. Furthermore, membrane processes were affected by the OMP treatment, involving stimulation of cutin biosynthesis, alteration of membrane lipids and an improved capacity for counteracting radical-mediated oxidative processes via the accumulation of phenolics and carotenoids. Several other stress-related compounds were affected by the OMP treatment, including polyamine conjugates, alkaloids and sesquiterpene lactones. Taken all together, OMP heightens this essential adaptation mechanism and increases tomato nutrient uptake and allocation, photosynthesis and plant performance under salt stress conditions thus improving resource use efficiency and tolerance to salinity. Although large-scale commercial application of OMP to mitigate plant salinity stress might currently not be economically viable, the present findings further corroborate the potential for development of a new class of formulations based on OMP analog molecules.

## Author contributions

GR: Had the original idea on testing omeprazole on vegetable crops and contributed in the set up of the experimental protocol; YR: Defined the scientific hypothesis, set up the experimental protocol, coordinated the research and he was significantly involved in writing; LL: Performed the whole the metabolomic analysis, and gave an important contribution on metabolomic results interpretation as well as a significant contribution in writing the manuscript; PC: Contributed in writing the physiological and ion analyses parts and run the PCA and heat map; AP and VC: Worked on the mineral and statistical analysis; CE-N: Was responsible for the greenhouse tasks; GC, MK, and SDP: Contributed in writing and improving the manuscript.

### Conflict of interest statement

The authors declare that the research was conducted in the absence of any commercial or financial relationships that could be construed as a potential conflict of interest.

## Supplementary material

Supplementary Table 1 Analysis of variance and mean comparisons for nitrate, phosphate, potassium, calcium, magnesium, sodium, and chloride in fruits of tomato plants grown under two salinity levels and treated with omeprazole (OMP) at three rates of application.

Supplementary Table 2 Raw data set as gained by UHPLC-ESI/QTOF-MS metabolomic analysis of tomato leaves under two salinity levels and three omeprazole (OMP) rates of application. Data are presented as raw intensities together with composite spectrum (accurate mass/ion abundance pairwise for each compound).

Supplementary Figure 1 Orthogonal Projections to Latent Structures Discriminant Analysis (OPLS-DA) on tomato leaves metabolome from plants grown under nonsaline (1 mM NaCl, upper pane) or saline nutrient solution (75 mM NaCl, lower pane), following OMP application at three rates (0, 10, or 100 μM). Individual replications are given in the class prediction model score plot.
