# Supplementary material for: Physiological and Metabolic Responses Triggered by Omeprazole Improve Tomato Plant Tolerance to NaCl Stress
Source: Front Plant Sci. 2018 Feb 27;9:249. doi: 10.3389/fpls.2018.00249 (PMC5835327; doi:10.3389/fpls.2018.00249)
Supplement: Supplementary Table 1 — Analysis of variance and mean comparisons for nitrate, phosphate, potassium, calcium, magnesium, sodium, and chloride in fruits of tomato plants grown under two salinity levels and treated with omeprazole (OMP) at three rates of application. [file Table1.DOC]

**Table S1.** Analysis of variance and mean comparisons for nitrate, phosphate, potassium, calcium, magnesium, sodium and chloride in fruits of tomato plants grown under two salinity levels and treated with omeprazole (OMP) at three rates of application.

| Source of variance | NO_3_^-^  (mg g^-1^ dw) | PO_4_^3-^  (mg g^-1^ dw) | K^+^  (mg g^-1^ dw) | Ca^2+^  (mg g^-1^ dw) | Mg^2+^  (mg g^-1^ dw) | Na^+^  (mg g^-1^ dw) | Cl^-^  (mg g^-1^ dw) |
| --- | --- | --- | --- | --- | --- | --- | --- |
| Salinity (S) | *** | ns | *** | ns | *** | *** | *** |
| Omeprazole (OMP) | ns | ns | ns | ns | * | ns | ns |
| S x O | ns | ns | ns | ns | ** | ns | ns |
|  |  |  |  |  |  |  |  |
| Salinity (mM NaCl) |  |  |  |  |  |  |  |
| 1 | 0.26 a | 11.8 | 40.14 a | 0.33 | 0.76 a | 0.76 b | 4.20 b |
| 75 | 0.10 b | 12.4 | 32.28 b | 0.34 | 0.56 b | 6.48 a | 18.47 a |
|  |  |  |  |  |  |  |  |
| Omeprazole (μM) |  |  |  |  |  |  |  |
| 0 | 0.18 | 12.3 | 36.32 | 0.29 | 0.58 b | 3.93 | 11.27 |
| 10 | 0.16 | 11.7 | 36.40 | 0.38 | 0.70 a | 3.44 | 11.45 |
| 100 | 0.19 | 12.3 | 35.92 | 0.33 | 0.69 a | 3.49 | 11.29 |
|  |  |  |  |  |  |  |  |
| S × OMP |  |  |  |  |  |  |  |
| 1 mM NaCl x 0 μM OMP | 0.27 | 11.7 | 40.16 | 0.31 | 0.59 b | 0.60 | 3.47 |
| 1 mM NaCl x 10 μM OMP | 0.22 | 11.4 | 41.01 | 0.33 | 0.81 a | 0.74 | 4.63 |
| 1 mM NaCl x 100 μM OMP | 0.29 | 12.4 | 39.26 | 0.35 | 0.87 a | 0.94 | 4.50 |
| 75 mM NaCl x 0 μM OMP | 0.10 | 12.0 | 32.47 | 0.28 | 0.58 b | 7.27 | 19.07 |
| 75 mM NaCl x 10 μM OMP | 0.11 | 12.0 | 31.80 | 0.44 | 0.58 b | 6.14 | 18.27 |
| 75 mM NaCl x 100 μM OMP | 0.09 | 12.3 | 32.58 | 0.32 | 0.51 b | 6.04 | 18.09 |

ns,*,**, *** Nonsignificant or significant at P ≤ 0.05, 0.01, and 0.001, respectively. Different letters within each column indicate significant differences according to Duncan’s multiple-range test (P = 0.05).
