# Supplementary material for: Physiological and Metabolic Responses Triggered by Omeprazole Improve Tomato Plant Tolerance to NaCl Stress
Source: Front Plant Sci. 2018 Feb 27;9:249. doi: 10.3389/fpls.2018.00249 (PMC5835327; doi:10.3389/fpls.2018.00249)
Supplement: Supplementary Figure 1 — Orthogonal Projections to Latent Structures Discriminant Analysis (OPLS-DA) on tomato leaves metabolome from plants grown under nonsaline (1 mM NaCl, upper pane) or saline nutrient solution (75 mM NaCl, lower pane), following OMP application at three rates (0, 10, or 100 μM). Individual replications are given in the class prediction model score plot. [file Image1.pdf]

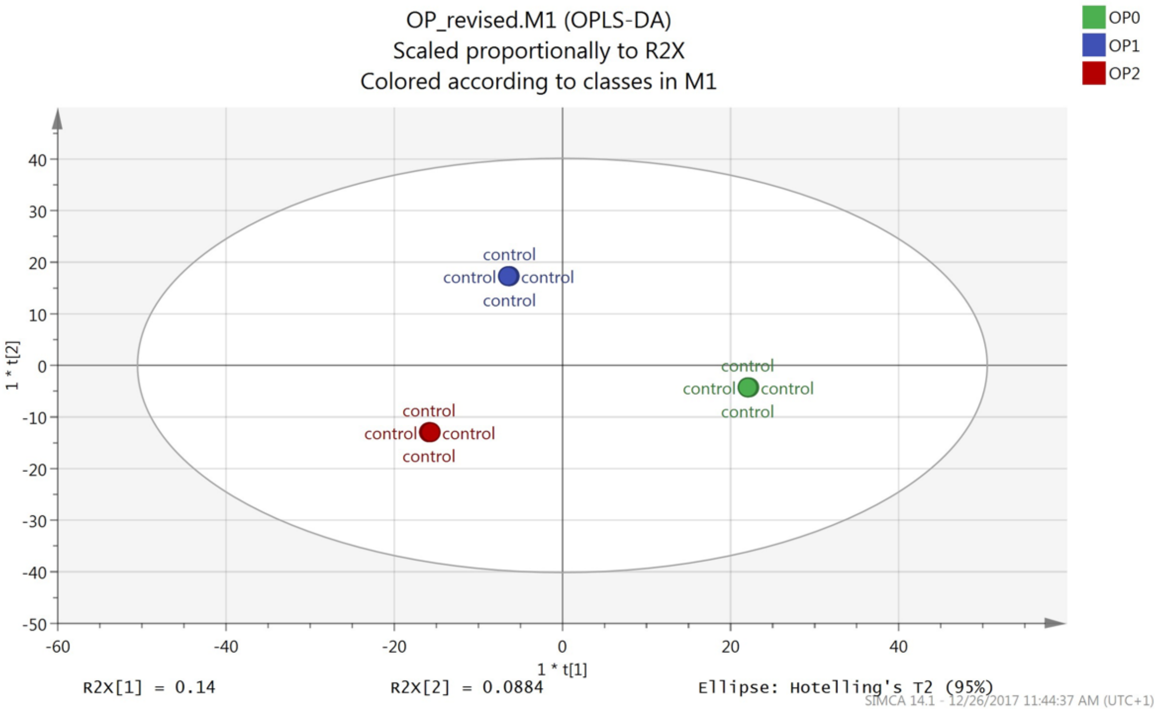
OP_revised.M1 (OPLS-DA)
Scaled proportionally to R2X
Colored according to classes in M1
OP0
OP1
OP2
control
control
control
control
1 * t[2]
1 * t[1]
R2X[1] = 0.14
R2X[2] = 0.0884
Ellipse: Hotelling's T2 (95%)
SIMCA 14.1 - 12/26/2017 11:44:37 AM (UTC+1)

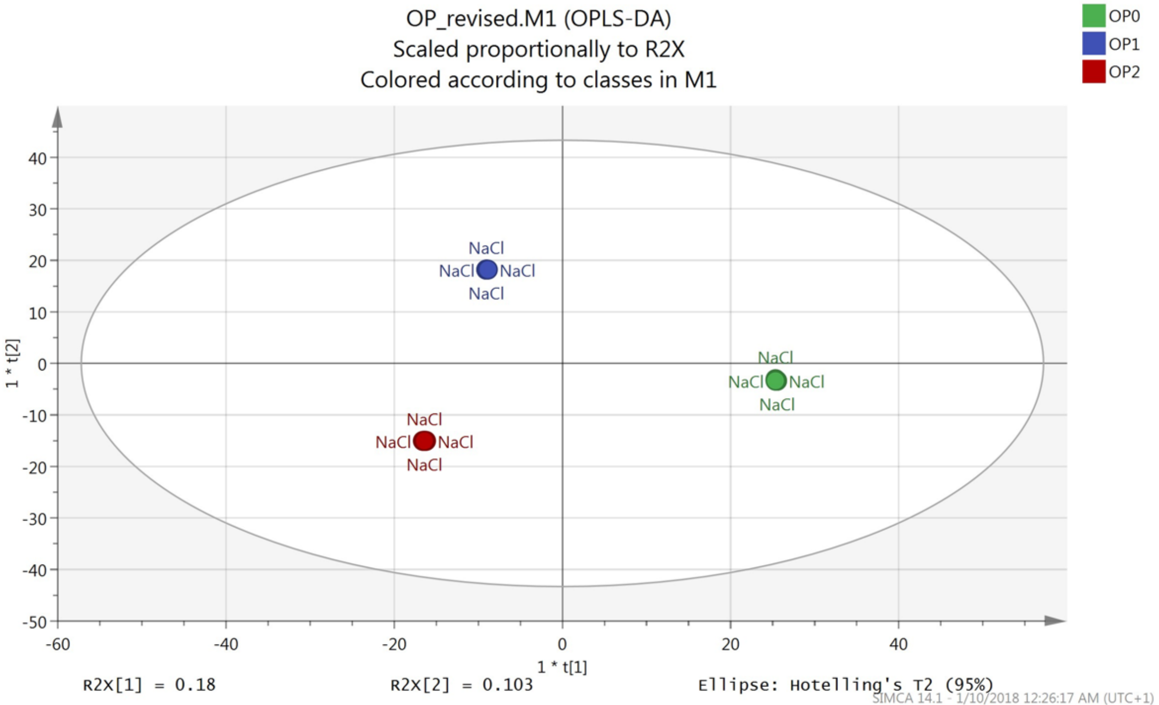
OP_revised.M1 (OPLS-DA)
Scaled proportionally to R2X
Colored according to classes in M1
OP0
OP1
OP2
NaCl
NaCl
NaCl
NaCl
1 * t[2]
1 * t[1]
R2X[1] = 0.18
R2X[2] = 0.103
Ellipse: Hotelling's T2 (95%)
SIMCA 14.1 - 1/10/2018 12:26:17 AM (UTC+1)
